# The diagnostic trajectory in autism and intellectual disability in Quebec: pathways and parents’ perspective

**DOI:** 10.1186/s12887-021-02864-0

**Published:** 2021-09-09

**Authors:** Mélina Rivard, Coulombe Patrick, Catherine Mello, Diane Morin, Marjorie Morin

**Affiliations:** 1 Université du Québec à Montréal, C.P. 8888 succursale Centre-ville, Montréal, Québec H3C 3P8 Canada; 2 D22 Data Consulting, Montréal, Canada; 3 The Pennsylvania State University – Berks, Reading, PA 16801 USA

**Keywords:** Assessment, Autism, Intellectual disabilities, Services trajectory, Quality, parent’s perspective

## Abstract

**Background:**

This paper aimed to describe the diagnostic service trajectory of families of children with autism or intellectual disability in the province of Québec and identify predictors of parents’ perceptions of its quality.

**Methods:**

The Evaluation of the services Trajectory in Autism by Parents instrument was completed by 259 parents at an assessment clinic. Children’s clinical records were also examined.

**Results:**

On average 26 months elapsed between their first concerns and their child’s diagnosis, a period during which few (25%) received support. Parents’ evaluations were generally positive but were lower for the accessibility of the pre-assessment phase and the flexibility of the assessment process. Longer delays and a greater number of professionals consulted were associated with lower quality ratings. Some language-, immigration status-, and income-related differences in families’ appraisals were noted.

**Conclusion:**

The diagnostic trajectory for neurodevelopmental disorders within public services in Québec presents some efficiency and accessibility challenges. Possible improvements are proposed to facilitate screening and to support families throughout this phase of their trajectory.

## Background

For parents of children with a neurodevelopmental disorders (see DSM-5) [1] such as autism spectrum disorder (ASD) and intellectual disability (ID) and global developmental delay (GDD), obtaining a diagnosis for their child is a particularly critical but challenging process: they must quickly develop an expertise in the relevant healthcare and social services systems and advocate for themselves to obtain answers to their concerns and, ultimately, the support and services their child needs [2]. Indeed, the structure of the healthcare and social services systems responsible for the evaluation and diagnosis of young children can have a powerful impact on their experiences, as navigating this system tends to be complex. Because a formal diagnostic label is often required to gain access to public intervention and support services, this process also time sensitive and will influence the future of children and families. Parents who have become aware of the importance of early intervention for their child’s prognosis experience a sense of urgency often coupled with helplessness as they confront systemic barriers to accessing a diagnosis rapidly [3, 4].

In the context of neurodevelopmental disorders, the concept of service trajectory refers to all the steps that families take to gain access to a range of services and interventions as well as their experience of these supports and the transitions between them [5, 6]. It is therefore a dynamic experience that encompasses both the objective characteristics of events or processes and their subjective appraisal by family members. Initiatives to improve referral and diagnosis service trajectories for neurodevelopmental disorders require a comprehensive description of families’ diagnostic pathways, from the moment they first suspect atypical development until they obtain a diagnosis, along with the barriers and facilitations they encounter along the way. It is also important to specifically consider how parents view this journey, as their perception of the quality of this experience can shape their relationship with healthcare and social services providers, their parenting practices at home, and their investment in evidence-based interventions following the diagnosis [4, 5, 7].

### Barriers to accessing a neurodevelopmental disorder diagnosis

Systemic barriers to accessing diagnostic services have been widely documented, particularly with respect to ASD and in the United States. These barriers contribute to the economic and psychosocial difficulties experienced by families of children with neurodevelopmental disorders [2–4]. For examples, families may face sizeable delays in obtaining a referral to evaluation services, a lack of continuity between providers, intricate administrative requirements to gain access to assessment, inconsistent or incomplete information about the diagnostic process or the diagnostic itself and the following adequate services, and the need to coordinate care across multiple providers and organizations [3–8].

In a recent study in the United Kingdom, the delay between parents’ first contact with a healthcare provider about their concerns regarding their child and receiving a diagnosis of ASD was approximately 3.5 years [9]. Families in the United States wait on average 2 years between first suspecting their child may have ASD and obtaining an official diagnosis, a time period that usually spans ages 2 through 4 for their child [2, 10]. Fewer studies have examined service accessibility in Canada, where the present study was conducted, but research thus far indicates that delays for an ASD diagnosis and subsequent access to early intervention may add up to 4 years [11].

Research conducted in the United States also suggests that systemic barriers disproportionately affect some segments of the population: there is evidence of racial, ethnic, and socioeconomic disparities in access to services and in the quality of services received [12–14]. Because diagnostic pathways are a function of the structure of prevailing healthcare and social systems as well as their broader sociocultural context, more studies are needed at a Canadian level to better understand families’ experiences with services trajectories and the factors that shape these. To this end, the present study was an in-depth analysis of the diagnostic service trajectory, as well as its evaluation by parents and the factors associated with its perceived quality, in the province of Quebec.

### Parents’ perspective on the diagnostic services trajectory

To date, parents’ perspective of the services trajectory has primarily been studied through the lens of satisfaction with specific services or providers. Overall, these studies describe a sense of dissatisfaction with the diagnostic process among parents of children with ASD [4, 9]. However, comparatively less attention has been paid to how parents appraise the quality of their overall diagnostic services trajectory in of the broader context of neurodevelopmental disorders (i.e., not limited to ASD). Furthermore, while the notion of accessibility has received some attention in the literature, few studies have systematically evaluated the quality of service trajectories across a range of determinants, as experienced by families: accessibility, continuity, validity, flexibility, and empathy-listening in provider-family relationships.

The present study sought to replicate, integrate, and build upon previous findings regarding the steps, barriers, and facilitators to obtaining a neurodevelopmental diagnosis on one hand, and data on parental satisfaction with diagnostic evaluation services on the other hand. It aimed examine the quality of services diagnostic trajectory in the larger context of neurodevelopmental disabilities, within the bilingual public healthcare and social services system of the province of Québec. Furthermore, it endeavored to do so through a structured framework, Evaluation of the services Trajectory in Autism by Parents (ETAP) [5], which supports the description of the diagnostic pathway and an evaluation of its quality from the perspective of primary stakeholders (i.e., parents). The specific goals of this project were to 1) document the steps parents took, their experiences, and the services they received; 2) assess their perceptions of specific quality determinants according to the ETAP model; and 3) identify systemic, family, and child-related factors associated with parents’ appraisal of the quality of each phase of their diagnostic trajectory.

## Method

This study was part of a longitudinal investigation of the service trajectories of families of children with ASD, (ID) or GDD throughout the early childhood period and leading up to their transition to school in Quebec (Canada). The study was approved by the ethics committees of [removed for blind review].

### Participants

The parents of 259 children referred to a free assessment clinic within the public healthcare and social services network in Montreal (largest city in Quebec, Canada) for a suspicion of ASD, ID or GDD participated in the study. In order for their parents to be eligible to participate in the study, their child had to be between 12 months to 5\ 11 months and to have been referred for ASD, ID or GDD assessment by a healthcare or social services provider. The label GDD refers to a significant (exceeding two standard deviations on standardized tests) in cognitive and adaptive skills domains observed before 5 years of age. GDD is the diagnosis given to a child when ID is suspected but, because of the child’s young age and various circumstances, the clinical team decided to use this temporary label and recommended reassessment when the child reaches school age.

Tables 1 and 2 present the sociodemographic characteristics of participating families. At the time they were diagnosed, children’s age ranged between 24 and 75 months, with a mean age of 48 months (*SD* = 13). Among them were 207 boys (80%) and 52 girls (20%). At the moment of the evaluation process, 91% of children were enrolled in daycare services.

**Table 1:** Overview of Family Characteristics

Variables	*n*	% Missing	%
Civil Status	**250**	3.5%	
1. Married or common-law partners	182		72.8%
2. Separated or divorced	16		6.4%
3. Stepfamily	12		4.8%
4. Single	40		16.0%
Annual household income (CAD)	**235**	9.3%	
1. $10,000-29,999	64		27.2%
2. $30,000-49,999	52		22.1%
3. $50,000-69,999	35		14.9%
4. $70,000-89,999	24		10.2%
5. $90,000-119,999	28		11.9%
6. $120,000-139,999	32		13.6%
Mothers’ level of education	**245**	4.6%	
1. High school or lower	57		23.3%
2. DCS / DVS^a^	64		26.1%
3. University	124		50.6%
Fathers’ level of education	**231**	10.8%	
1. High school or lower	56		24.2%
2. DCS / DVS	57		24.7%
3. University	118		51.1%
Mothers’ occupation	**249**	3.9%	
1. Full-time, salaried employee	88		35.3%
2. Part-time, salaried employee	39		15.7%
3. Contract worker	5		2.0%
4. Homemaker	94		37.8%
5. Student	17		6.8%
6. Other (on leave, retired)	6		2.4%
Fathers’ occupation	**233**	10.0%	
1. Full-time, salaried employee	180		77.3%
2. Part-time, salaried employee	10		4.3%
3. Contract worker	2		0.9%
4. Homemaker	19		8.2%
5. Student	12		5.2%
6. Other (retired, unemployed, imprisoned)	10		4.3%
Mothers’ place of birth	**249**	3.9%	
1. Canada	72		28.9%
2. United States	1		0.4%
3. Central or South America	41		16.5%
4. Africa	75		30.1%
5. Asia and Middle East	41		16.5%
6. Europe	17		6.8%
7. Oceania	2		0.8%
Fathers’ place of birth	**242**	6.6%	
1. Canada	63		26.0%
2. United States	3		1.2%
3. Central or South America	38		15.7%
4. Africa	80		33.1%
5. Asia and Middle East	48		19.8%
6. Europe	9		3.7%
7. Oceania	1		0.4%
Language spoken at home	**249**	3.9%	
1. French (with or without other languages)	149		59.9%
2. English (with or without languages, except French)	52		20.9%
3. Other	48		19.3%
Presence of mental health diagnoses in siblings	**239**	7.7%	
No	181		75.7%
Yes	58		24.3%
Presence of mental health diagnoses for the mother	**245**	5.8%	
No	216		88.1%
Yes	29		11.9%
Presence of mental health diagnoses for the father	**246**	5.0%	
No	207		84.2%
Yes	39		15.9%

*Note*. ^a^ In Quebec, the diploma of collegiate studies (DCS) is a postsecondary degree in preparation for university-level education or a trade; the diploma of vocational studies (DVS) is a secondary degree in preparation for a specialized occupation

**Table 2:** Descriptive Statistics for Child Variables

Variables				*n*	Missing	%
Child’s primary diagnosis				**259**	0.0%	
1. Autism spectrum disorder				142		54.8%
2. Global developmental delay or intellectual disability				9		3.5%
3. Autism spectrum disorder and global intellectual delay or intellectual disability				76		29.3%
4. Other (e.g., language disorders, attention deficit/hyperactivity disorder, neurodevelopmental disorder requiring reevaluation)				32		12.4%
	*n*	Missing	*M*	*SD*	Range	
ABAS GAC	248	4.3%	72.1	16.1	40–114	
CBCL Total Problems	243	6.2%	60.1	12.5	35–93	

*Note*. *ABAS GAC* Adaptive Behavior Assessment System, Global Adaptive Composite score, *CBCL* Child Behavior Checklist. In Quebec (Canada)

### Measures

#### Clinical records

Clinical records included the child’s assessment results and parents’ responses to questionnaires administered by the clinic as part of the diagnostic evaluation process. Global scores for challenging behavior and emotional difficulties (Total Problems score of the Child Behavior Checklist for Ages 1.5–5) [15] and for adaptive behavior (global adaptive composite of the Adaptive Behavior Assessment System-II, Preschool Version) [16] were retained as general indicators of children’s clinical profile.

#### Services trajectory evaluation

The diagnostic version of the ETAP questionnaire (known as ETAP-1) [5] is an instrument in which parents or other caregivers provide factual and subjective information on the steps they took from the moment they first suspected atypical development in their child until they received a formal diagnosis. It consists of 65 questions organized into five sections. Sections 1 and 2 cover the period leading up to the child’s diagnostic evaluation (Phase 1: pre-assessment). Sections 3 and 4 cover the evaluation process itself (Phase 2: assessment and diagnosis), and Section 5 relates to this entire period (i.e., Phases 1 and 2 taken together). Sections 1 and 3 collect descriptive information about Phase 1 and 2, respectively, through open-ended and multiple questions about the steps taken by the family, the time elapsed between each step, and the professionals than were consulted at that time. In Sections 2, 4, and 5, respondents are asked to rate the quality of each portion of their services trajectory (Phase 1 and 2, and overall) according to five quality determinants identified in previous research: accessibility, continuity, flexibility, validity, and empathy-listening skills [5].

Accessibility refers to the family’s ability to obtain the services and support they need at a specific point of their trajectory. Continuity describes the degree to which services were provided in a coherent, coordinated sequence. Flexibility refers to services or service providers’ ability to adapt to meet the needs of the child or the family at a given moment. In contrast, validity pertains to respondents’ perceptions that the services and information they receive are relevant and adequate for the child’s clinical profile and needs. Finally, empathy-listening skills refer to providers’ ability to respond adequately to the concerns and needs expressed by the family.

The items in these Section 2, 4, and 5 are rated on a 5-point (1 = *strongly disagree*, 5 = *strongly agree*) for which higher numbers are consistent with higher perceived quality. The structure of the instrument (five determinants assessed in two distinct phases) was confirmed through factor analyses. Additionally, the instrument showed excellent internal consistency, Cronbach’s α = .95; it also demonstrated specificity and convergent validity (*r* = .73) with a provider-specific measure of satisfaction, discriminant validity with family quality of life with related measures, and sensitivity to differences in service delivery models [5].

#### Procedure

Once their child had received a diagnosis, a member of the clinic’s staff provided parents with a form describing the study on which they could indicate their interest to be contacted by the research team. Those who did so met with a research assistant at the clinic or at the family residence. This interview began with written, informed consent procedures. Then, participants completed the background information questionnaire (5–10 min) and completed the ETAP questionnaire (30–45 min).

#### Analysis

Paired samples *t*-tests, using Bonferroni’s correction to maintain a .05 familywise error rate, were used to compare mean quality appraisals during the pre-assessment and diagnostic evaluation phases. To examine whether these appraisals varied as a function of systemic, family, and child variables, we estimated regression models separately for each phase and overall using path analysis with full-information maximum likelihood (FIML) estimation in order to include cases with missing data. Ordinal variables (household income and parents’ education) were treated as scale variables. Categorical predictors were coded using deviation effect coding: the interpretation of each coefficient is the difference in the average evaluation for a given category and the overall (sample) mean. Where necessary, sparse categories were combined with similar categories to address singularity concerns.

## Results

Table 3 summarizes key information about families’ diagnostic trajectory (e.g., child’s age at diagnosis, number of professionals met during the process). Table 4 provides details on the origin and nature of parents’ first concerns, and with whom families first consulted about these concerns. Parents had their first concerns about their child’s development around 22 months (mostly about language delay); these were mostly raised by the mother, but also daycare educators and healthcare providers. On average 26 months elapsed between these concerns and a formal diagnosis (mean age of 48 months). Throughout the entire diagnostic trajectory, 77% of families said they received information pertaining to their child’s (suspected or official) diagnosis and appropriate services. Fewer than 25% of families said they received support or intervention services (only occasional, on-demand support, e.g., speech-language therapy) prior to receiving a formal diagnosis; these were primarily services obtained through private providers, for which families paid out of pocket.

**Table 3:** Descriptive Statistics for Quantitative Systemic Variables

Variable	*n*	% Missing	*M*	*SD*	Range
Age of the child at the time of first concerns	240	7.3%	22.3	9.9	0^a^-60
Waiting time for a consultation about first concerns	211	18.5%	2.7	5.0	0–36
Age of the child at the time of diagnosis	259	0.0%	48.0	12.9	23.8–75.4
Time elapsed between first concerns and diagnosis	241	7.0%	26.2	11.2	3.2–58.1
Time elapsed between first concerns and initial assessment	259	0.0%	3.2	1.5	0.7–13.9
Number of providers consulted prior to assessment	244	5.8%	3.2	1.5	0–7
Number of providers consulted at the assessment clinic	241	7.0%	3.5	1.1	1–6
Total number of providers consulted prior to the diagnosis	247	4.6%	6.6	2.0	2–12

*Note*. Children’s age and waiting periods are specified in months. ^a^ In three cases, concerns emerged in the first two months of the child’s life, in relation to a developmental disorder diagnosis in an older sibling

**Table 4:** Families’ First Concerns About Their Child’s Development

	*n*	% Missing	%
**Source of first concerns**	**211**	18.5%	
**Family members**	**141**		**66.8%**
Mother only	94		44.5%
Father only	7		3.3%
Both parents	31		14.7%
Other relative	9		4.3%
**Non-family members**	**70**		**33.2%**
Daycare educator	31		14.7%
Pediatrician or family physician	26		12.3%
Other medical provider	13		6.2%
**Nature of first concerns**	**246**	5.0%	
Language delay	156		63.4%
Atypical motor development	32		13.0%
Atypical social development	28		11.4%
Challenging behavior	20		8.1%
Other concerns	10		4.1%
**Initial consultation about first concerns**	**244**	5.8%	
Pediatrician	126		51.6%
Family physician	47		19.3%
Speech-language pathologist	19		7.8%
Other healthcare provider	52		21.3%

### Quality of the trajectory: determinants and phases

Table 5 shows the scores for each of the five quality determinants of the trajectory evaluation for the pre-assessment phase (Phase 1) and the assessment and diagnosis phase (Phase 2), as well as change in these determinants between those two phases. Overall, for Phase 1 parents had generally positive (i.e., higher than the neutral point, 3) evaluations across determinants, with Accessibility being rated lowest (*M =* 3.7, *SD =* 1.0) and Empathy receiving the highest ratings (*M* = 4.2, *SD* = 0.8). All pairwise comparisons between determinants for this phase were significant except between Validity and Accessibility, and between Validity and Flexibility. Parents also rated Phase 2 positively; Flexibility was rated the lowest (*M* = 4.3, *SD =* 0.7) and Empathy was rated the highest (*M* = 4.6, *SD =* 0.7). For this phase, Validity was only rated significantly lower than Empathy, Empathy was rated significantly higher than all other determinants, and Flexibility was rated significantly lower than all determinants except Validity. Importantly, the ratings given to each determinant were significantly higher for the diagnosis phase compared to the pre-assessment phase.

**Table 5:** Ratings for Quality Determinants of the Pre-Assessment and the Assessment and Diagnosis Phases of Families’ Service Trajectory

Determinant	Phase 1: Pre-Assessment						Phase 2: Assessment and Diagnosis						Change	
	Frequencies			Mean	SD	Subsets	Frequencies			Mean	SD	Subsets		
	Negative	Neutral	Positive				Negative	Neutral	Positive					*d*
Continuity	7%	0%	93%	4.0	0.7	a	2%	0%	98%	4.4	0.6	a	0.4***	.54
Access	20%	9%	71%	3.7	1.0	b	3%	2%	95%	4.4	0.7	a	0.7***	.70
Flexibility	14%	11%	75%	3.8	0.9	c	3%	7%	91%	4.3	0.7	b	0.5***	.48
Validity	16%	7%	77%	3.8	1.0	b,c	4%	4%	91%	4.4	0.8	a,b	0.6***	.47
Empathy	5%	9%	86%	4.2	0.8	d	2%	4%	94%	4.6	0.7	c	0.4***	.41

*Note*. Frequency distributions are organized as follows: negative ratings (below 3), neutral (3), and positive (above 3). All tests were paired-samples *t*-tests with *df* = 240–245 with the corresponding effect size (Cohen’s *d*). For within-phase comparisons, the Bonferroni-corrected α for 10 related tests is .05/10 = .005. Two dimensions that share a subset do not differ; two dimensions that do not share any subsets differ significantly. For tests of change between phases, the Bonferroni-corrected alpha for 5 related tests is .05/5 = .01

*** *p* < .001

Finally, the ETAP questionnaire also includes a section which invites parents to provide a general appraisal of the quality of the services trajectory for the whole diagnostic period, from the first suspicion to disclosure of the diagnosis (see Table 6). Accessibility was rated as having the lowest quality throughout their trajectory (*M* = 3.5, *SD* = 1.2) and Empathy as having the highest quality (*M* = 4.3, *SD* = 0. 8). All pairwise comparisons between determinants attained significance except between Continuity and Flexibility, and between Validity and Empathy.

**Table 6:** Ratings for Quality Determinants for the Overall Diagnostic Evaluation Trajectory

Global Assessment	Frequencies			*M*	*SD*	Subsets
	Negative	Neutral	Positive			
Continuity	13%	8%	79%	4.0	1.0	a
Access	25%	16%	59%	3.5	1.2	b
Flexibility	10%	18%	72%	3.9	1.0	a
Validity	6%	11%	84%	4.2	0.8	c
Empathy	3%	10%	87%	4.3	0.8	c

*Note*. Frequency distributions are organized as follows: negative ratings (below 3), neutral (3), and positive (above 3). All tests were paired-samples t-tests with *df* = 241–242, the Bonferroni-corrected α for 10 related tests is .05/10 = .005. Two dimensions that share a subset do not differ; two dimensions that do not share any subsets differ significantly

### Factors associated with quality of the trajectory

Table 7 displays the results of the regression models predicting global quality ratings (averaged across the five determinants) for each of the two phases, as well as ratings for each quality determinant in parents’ overall appraisal of their trajectory. Except language spoken at home (French), all significant predictors for the global quality evaluation of Phase 1 related to systemic variables. Several family characteristics and children’s problem behaviors were predictive of quality for Phase 2. A combination of systemic, family, and child variables accounted for each quality determinant of the overall trajectory.

**Table 7:** Regression Analyses Predicting Overall Quality and Specific Quality Determinants of the Diagnostic Evaluation Trajectory

Predictor	Phase 1	Phase 2	Overall Trajectory				
			Continuity	Access	Flexibility	Validity	Empathy
**Systemic**							
Age at first concern	−1.57	.44	−.34	−.65	−1.33	− 1.49	− 1.85*
Age at diagnosis	2.14*	−.58	.48	1.05	1.83	1.98	2.55*
Time elapsed between first concerns and diagnosis	−1.99*	.44	−.56	−.97	− 1.67	− 1.84*	−2.31*
Number of professionals consulted	−.13*	.09	−.01	−.02	−.04	.00	−.05
Receiving information on autism	.12	.25*	.12	.08	.12	.17*	.06
Receiving punctual services (mostly private)	.00	.07	−.13	−.15*	−.02	−.00	.04
**Family**							
Household income	−.13	−.36*	−.27*	−.10	−.20*	−.29*	−.25*
Number of siblings	.11	.01	.13	.08	.20*	.17*	.10
Sibling(s) also diagnosed	.11	.02	−.06	.09	−.08	−.20*	−.10
Non-nuclear family	.13	.00	.17*	.01	.15	.07	.15*
Mothers’ education	.03	.07	.02	−.06	.09	.11	.05
Fathers’ education	.05	.14	.09	.06	−.02	−.01	.03
Mental health diagnosis in father	−.07	.00	−.00	.12	−.12	.01	−.02
Mental health diagnosis in mother	.15	.04	.31*	.10	.18	.09	.13
Mothers’ immigrant status	−.10	.02	.11	−.05	−.03	−.07	.01
Fathers’ immigrant status	.12	−.20*	−.04	.15	−.04	.07	−.10
Mothers’ unemployment	−.13	−.01	−.08	.01	−.09	−.08	−.09
Fathers’ unemployment	−.05	−.22*	−.05	.04	.09	.07	−.01
Language spoken at home							
French (with or without other languages)	.20*	.16	.06	−.12	−.05	.02	.28*
English (with or without other languages, except French)	−.09	.04	.03	.21	−.06	.04	−.17
Other	−.11	−.20	−.09	−.09	.11	−.06	−.12
**Child**							
ABAS GAC score	.13	.05	−.07	.07	−.05	.02	.02
CBCL Total Problems score	.06	.19*	−.08	−.12	.01	−.00	.04
Diagnosis							
ASD	−.13	−.02	.03	.06	.02	−.15	.05
GDD or ID	.19	.09	−.05	−.40	.20	−.03	.06
ASD and GDD or ID	−.06	−.02	.02	.06	−.28*	.07	−.18
Other	−.01	−.05	−.00	.27	.06	.10	.07

*Note*. Table entries are *β* (standardized) regression coefficients estimated separately for each outcome (column), using all predictor variables as covariates. *ABAS GAC* Adaptive Behavior Assessment System, Global Adaptive Composite score, *CBCL* Child Behavior Checklist, *ASD* Autism spectrum disorder, *GDD* Global developmental delay, *ID* Intellectual disability

* *p* < .05

## Discussion

The complex and often fragmented structure of health and social services systems in Canada and elsewhere can entail unintended negative consequences and costs (in time and effort) for the families who must navigate these to access appropriate supports for their child [7, 11]. The present study therefore sought to elucidate the experiences of families in the province of Québec throughout their diagnostic trajectory in order to identify ways of facilitating this trajectory and better supporting them. Although these results are grounded in the structure of the public health and social services systems of Québec as well as its sociocultural context, a number of observations made in the present study are consistent with observations made in other regions and countries, such as the barriers in accessibility of diagnostic and the sociodemographic factors associated to those barriers.

### From parents’ first concerns to the diagnosis

The mean age of the child when parents first expressed concerns about atypical development (22 months) and the time elapsed between these concerns and the child receiving a diagnosis (26 months) are generally consistent with observations in other countries such as the United States and the United Kingdom [9, 10]. Furthermore, the present study’s results indicate that such delays are associated with a more negative appraisal of the quality of the service trajectory by parents. These observations are consistent with earlier studies reporting several negative impacts of this service gap and waitlists on child development and family adjustment [11, 17]. Yet only 25% of families in the present sample had received any form of services during the period leading up to the diagnosis; typically, these were one-time consultations, e.g., with a speech-language pathologist in a private practice. Taken together, these findings suggest the importance of improving access to diagnostic evaluation and, in parallel to this process, assisting families as soon as concerns are confirmed by key persons [18].

### Early symptoms and concerns

Mothers were most often the first person to raise concerns about the child’s development (typically in relation to language acquisition), with daycare educators coming in second. In the present study, 91% of children were enrolled in daycare; their educators were consulted as part of the assessment clinic’s evaluation process. This underscores the importance of educators’ as a significant person in children’s lives and their ability to observe children in a range of critical situations, such as in interactions with peers. Daycare educators develop a significant expertise and experiential knowledge of children’s development through their contact with them, and could thus be involved in systematic screening initiatives using a standardized instrument to identify children who may have atypical or delayed development [19, 20]. However, it is also crucial to consider means of screening children who do not make use of daycare services, for instance during routine immunizations or wellness visits with a family physician or pediatrician. Primary healthcare providers are usually parents’ first point of contact and a trusted source of advice when they suspect their child is developing atypically. Yet many parents feel as though these practitioners dismiss or minimize their concerns and tend to advocate for a “wait and see” approach, which could delay access to the child’s diagnosis [2]. The parents who participated in the present study evaluated the pre-assessment phase, which included the acknowledgment of parents’ concerns along with the screening and referral process, less positively than the subsequent phase, during which they interacted with the professionals who assessed their child, received a diagnosis, and were oriented toward relevant services. We suspect the gap in quality appraisals between these two phases as evaluated by ETAP is partly attributable to a lack of systematic screening procedures by frontline professionals in primary care settings, as well as insufficient concrete responses to parents’ concerns and desire for solutions.

The findings of the present study underscore the need to systematically include guidelines for universal screening for ASD during early well-child visits as part of healthcare providers’ initial training and continuing education curriculum [3]. Indeed, studies have identified substantial delays in the adoption of formal screening tools by healthcare professionals, many of whom report feeling insufficiently trained to use these [3]. These observations are concerning inasmuch as pediatricians and family physicians are often the first professionals with whom parents share their concerns, and on whom they rely for guidance. It has been suggested that first line health care providers could adopt a more proactive response to parents’ concerns by prompting initiating referrals to specialists for further developmental testing, and also by providing families with psychosocial support and connecting them with a wide range of resources in the community [4, 21]. Considering the present bottlenecks in access to specialists and assessments in Québec, it is also important to develop (and systematize referrals to) direct support and intervention alternatives that could be available, within the public system, before a diagnosis is made. This may ease healthcare providers’ concerns about the impact of a positive screening result on the family [22]. Delays in accessing evidence-based interventions for ASD and IDD have been documented worldwide and could have crucial implications for children’s prognosis. Innovative responses to this concern may entail simultaneously referring families for early intervention and parent support before a formal diagnosis is made [11, 18]. During the early stages of a child’s development, pediatricians and other primary care providers need to be aware of the importance of timely screening and referrals, as well as families’ need for support as they embark on the complex journey associated with their child’s condition. Their empathy, their ability to provide them with guidance and information they seek, and their presence as a source of continuity through these changes is invaluable to parents.

### A complex, interdisciplinary process

On average, parents had to consult more than six professionals before obtaining a diagnosis for their child; a larger number of professionals consulted was associated with lower perceived quality of the services trajectory. Coordinating multiple appointments, referrals, etc. is a burdensome process for parents, who must also contend with this discontinuity in services by repeating the same emotionally laden information to multiple individuals and completing redundant forms. Parents nevertheless appreciate that several experts, across multiple disciplines, collaborate to assess their child (Redacted for blind review, 2020). This experience could be facilitated by a care navigator who could ensure informational continuity and provide parents with support and guidance throughout the referrals and consultations [23].

### Appraisal of the quality of the diagnostic trajectory

In contrast with earlier studies suggesting high levels of dissatisfaction with the diagnostic process [7], participants had a positive perception of both phases of their diagnostic trajectory. It should be noted that ETAP does not measure satisfaction with specific services, but a more global appraisal of the quality of families’ entire trajectory according to five determinants. This multidimensional assessment provides a more nuanced understanding of families’ perspectives and can help to identify unmet needs and recognize organizational strengths. Specifically, accessibility was the least favorably perceived aspect of the overall diagnostic trajectory and, specifically, the pre-assessment phase. In contrast, empathy was rated highly throughout the diagnostic trajectory. Having a positive relationship with first-line providers who are seen as empathetic, trustworthy, and receptive to their perspective was previously identified as an important factor in parents’ satisfaction with services [2, 5, 21].

### Equity and access disparities

A substantial proportion of families included at least one parent who was born outside of Canada (71% of mothers and 74% of fathers, compared to 34% in the Montréal community) [24]. Although less representative of the broader community in which the study took place, this reflects the demographic makeup of families who had been on a waiting list for ASD assessment in the public sector. Immigration-, race-, and ethnicity- related disparities in access to evaluation and early intervention services have been noted on multiple occasions [12–14]. In the present study, families who spoke languages other than French at home or in which the father had immigrated to Canada reported lower-quality trajectories. Families whose child had a more complex clinical profile (i.e., a dual diagnosis of ID and ASD) also perceived a lower quality of their trajectory, specifically with respect to its flexibility [25]. These characteristics could have compounding impacts on families’ experiences, e.g., if they must navigate an unfamiliar system, while not fluent in the dominant language spoken within it, to obtain multiple services for a child with more complex needs. One counter-intuitive finding in the present study was that families with lower household incomes tended to rate the quality of their trajectory higher. This may be due to the fact that families who lacked the means to seek out private services (to alleviate the gap in support during waiting periods) are more likely to appreciate diagnostic evaluation services when they finally receive these.

### Generalizability of findings

The findings of this study are circumscribed to the context in which data were collected, a specialized diagnostic evaluation clinic in Québec (Canada). However, these provide pathways to solutions relevant not only to the province’s public network but also to other regions and countries where major challenges in terms of quality (including, but not limited to, accessibility) and equity exist with respect to diagnostic evaluation for neurodevelopmental disorders.

### Contributions to knowledge

**What does this study add to existing knowledge?**

- This study is one of the first to use a systematic framework to provide both a factual description of the diagnostic evaluation pathway for neurodevelopmental disorders and an overview of the subjective appraisal of its quality by service users, based on five empirically validated dimensions.
- This study acknowledges the relevance of service users’ perceptions and experiences as in identifying areas for improvement, and relates these to previously documented (e.g., delays) and novel (e.g., dual diagnoses) systemic and family-related factors.

**What are the key implications for public health interventions, practice or policy?** This study uses the ETAP instrument, which is available free of charge to researchers and service providers, in French and in English at this address (https://chaireditc.uqam.ca/etap/).

- It is suited to assess existing strengths and weaknesses of the diagnostic trajectory as implemented in different systems and contexts.
- The identification of accessibility as the primary challenge perceived by parents indicates the importance of streamlining screening and referral procedure as well as the need to support families as soon as concerns are raised.
- The provision of personalized guidance to families throughout the diagnostic trajectory may help to address concerns of equity, continuity, and flexibility.

## Data Availability

The datasets used and analysed during the current study are available from the corresponding author on reasonable request.

## Abbreviations

ASD: Autism spectrum disorder
ID: Intellectual disability
GDD: Global developmental delay
ETAP: Evaluation of the services Trajectory in Autism by Parents
